# Supplementary material for: Microscopic theory of colour in lutetium hydride
Source: Nat Commun. 2023 Nov 14;14:7360. doi: 10.1038/s41467-023-42983-z (PMC10646004; doi:10.1038/s41467-023-42983-z)
Supplement: Supplementary file 1 — Supplementary Information [file 41467_2023_42983_MOESM1_ESM.pdf]

Supplementary Information for
"Microscopic theory of colour in lutetium hydride"

Sun-Woo Kim,[1,*] Lewis J. Conway,[1,2] Chris J. Pickard,[1,2] G. Lucian Pascut,[3] and Bartomeu Monserrat[1,4,†]

[1]*Department of Materials Science and Metallurgy, University of Cambridge, 27 Charles Babbage Road, Cambridge CB3 0FS, United Kingdom*

[2]*Advanced Institute for Materials Research, Tohoku University, 2-1-1 Katahira, Aoba, Sendai 980-8577, Japan*

[3]*MANSiD Research Center and Faculty of Forestry, Stefan Cel Mare University (USV), Suceava 720229, Romania*

[4]*Cavendish Laboratory, University of Cambridge, J. J. Thomson Avenue, Cambridge CB3 0HE, United Kingdom*

Contents

Supplementary Note 1. Electronic band structure 3

Supplementary Note 2. Electron correlation 5

Supplementary Note 3. Electron-phonon coupling 6

Supplementary Note 4. Lutetium-hydrogen binary system 9

4.1. Crystal structures of LuH_2 and LuH_3 9

4.2. Reflectivity and colour of LuH_2 and LuH_3 10

4.3. Reflectivity and colour of nitrogen doped cubic LuH_2 and LuH_3 12

Supplementary Note 5. Lutetium-hydrogen-nitrogen ternary system 14

5.1. Crystal structures 14

5.2. Reflectivity and colour 15

Supplementary Note 6. Phonon dispersion 17

Supplementary Note 7. Phonon-mediated superconductivity 19

Supplementary Note 8. Details of photoreliastic rendering 20
8.1. Benchmark tests on Lu and LuH_2 20
8.2. Surface roughness effect 20
8.3. Choice of exchange-correlation functional 21
Supplementary Note 9. Convergence tests on reflectivity 23
Supplementary References 23

Supplementary Note 1. ELECTRONIC BAND STRUCTURE

We show the electronic band structure and density of states of $Fm\overline{3}m$ LuH_2 in Fig. S1**a** calculated using semilocal DFT in the PBEsol approximation (DFT model in the main text). In agreement with earlier calculations, we find a metallic band structure with the Fermi level crossing highly dispersive bands that lead to an overall small density of states at the Fermi energy.

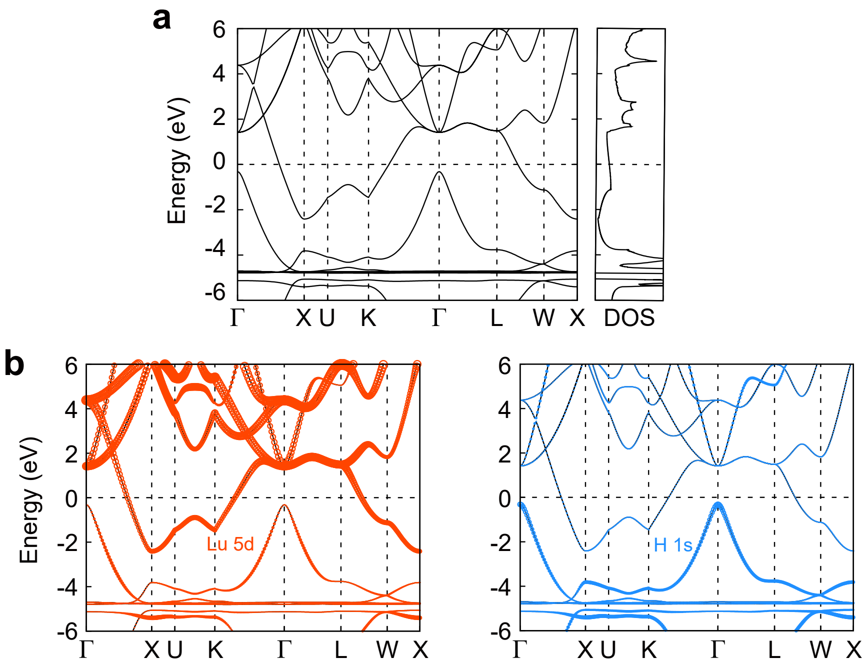

FIG. S1: **Electronic structure of pristine LuH_2. a.** Band structure and density of states of pristine LuH_2. **b.** Orbital-projected band structure of LuH_2. The band structure is projected onto lutetium 5*d* (left) and hydrogen 1*s* (right) orbitals and the size of the open circles is proportional to the projected weight.

We also show the electronic band structure projected on various lutetium and hydrogen atomic orbitals in Fig. S1**b**. The states derived from the lutetium 5*d* orbitals are highly dispersive.

Figure S2 depicts the evolution of the band structure of LuH_2 under pressure. Most bands only show rigid shifts with increasing pressure, apart from the bands near the X point in the Brillouin zone which exhibit a more significant re-organisation.

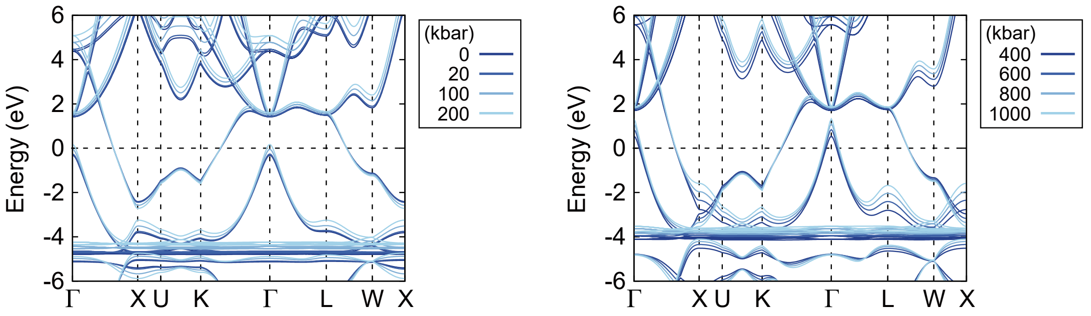

FIG. S2: **Pressure dependence of the electronic band structure of LuH_2.**

Supplementary Note 2. ELECTRON CORRELATION

Lutetium has a partially filled $5d$ shell which may lead to strong electronic correlations. In Fig. S3 we show the electronic band structure of LuH_2 calculated using DFT corrected with a Hubbard U term (DFT+U model in the main text). We show calculations using U values in the range from 0 to 5 eV. The band structure changes with U are small, which is consistent with the small changes reported in the main text for the reflectivity and colour. Similarly, we have tested the role of dynamical correlations using DFT augmented with dynamical mean field theory, and find negligible contributions.

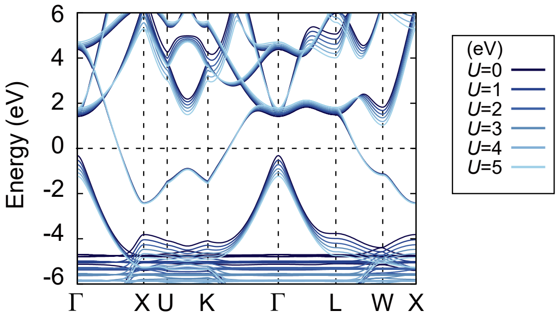

FIG. S3: **Dependence of the electronic band structure of LuH_2 on the Hubbard U parameter.**

Supplementary Note 3. ELECTRON-PHONON COUPLING

Hydrogen is the lightest of all elements, and as such it exhibits significant quantum fluctuations which are responsible for the large electron-phonon coupling driving high temperature superconductivity in high pressure hydrides. In the main text, we present the reflectivity of LuH_2 under ambient conditions including the effects of electron-phonon interactions arising from quantum and thermal fluctuations. In this Supplementary Note we present additional details about the associated calculations.

As described in the main text, the dielectric function at temperature T renormalized by electron-phonon coupling is given by [1, 2]:

$$\varepsilon_2(\omega;T) = \frac{1}{\mathcal{Z}} \sum_{\mathbf{s}} \langle \Phi_{\mathbf{s}}(\mathbf{u}) \left| \varepsilon_2(\omega;\mathbf{u}) \right| \Phi_{\mathbf{s}}(\mathbf{u}) \rangle \, e^{-E_{\mathbf{s}}/k_{\mathrm{B}}T}, \tag{1}$$

where $\mathcal{Z}$ is the partition function, $|\Phi_{\mathbf{s}}(\mathbf{u})\rangle$ is a harmonic eigenstate $\mathbf{s}$ of energy $E_{\mathbf{s}}$, $\mathbf{u} = \{u_{\mathbf{q}\nu}\}$ is a vector containing all atomic positions expressed in terms of normal mode amplitudes $u_{\mathbf{q}\nu}$, and k_{B} is Boltzmann's constant.

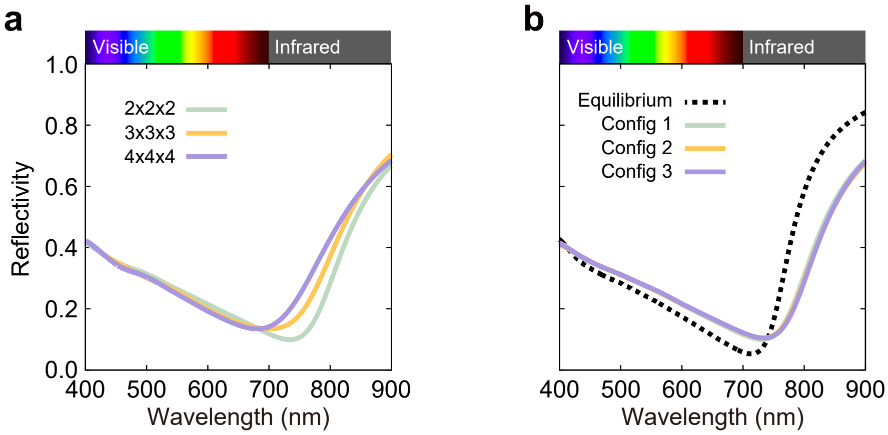

FIG. S4: **Convergence of electron-phonon coupling effects on the reflectivity of LuH_2.** **a.** Convergence with respect to supercell size. **b.** Convergence with respect to the number of stochastic configurations in the Monte Carlo integration.

We evaluate Eq. (1) using Monte Carlo integration accelerated with thermal lines [3]. We start from the phonon calculations described in Supplementary Note 5 and we then generate atomic configurations in which the atoms are distributed according to the harmonic nuclear

wave function in which every normal mode has an amplitude equal to:

$$u_{\mathbf{q}\nu} = \pm \left(\frac{1}{2\omega_{\mathbf{q}\nu}} \left[1 + 2n_{\mathrm{B}}(\omega_{\mathbf{q}\nu}, T) \right] \right)^{1/2}, \tag{2}$$

where $n_{\mathrm{B}}(\omega, T)$ is the Bose-Einstein factor, and the sign of the amplitude is chosen stochastically. These calculations involve two convergence parameters which need to be tested. First, electron-phonon interactions need to be converged with respect to supercell size (or equivalently with respect to Brillouin zone grid size). Figure S4**a** shows the electron-phonon renormalised reflectivity calculated using increasingly large supercell sizes. There is some dependence on supercell size, but the overall reflectivity shape is relatively consistent between them. In the main text we use the results from the $4 \times 4 \times 4$ calculations. Second, in the Monte Carlo integration, the electron-phonon renormalised dielectric function is in principle obtained by averaging over stochastic configurations, where the number of configurations is a convergence parameter. We find that the electron-phonon renormalised reflectivity converges very rapidly with respect to the number of configurations included in the calculation: Fig. S4**b** shows three different configurations (compared to the equilibrium configuration) and the curves are indistinguishable. We use a single configuration for the results reported in the main text.

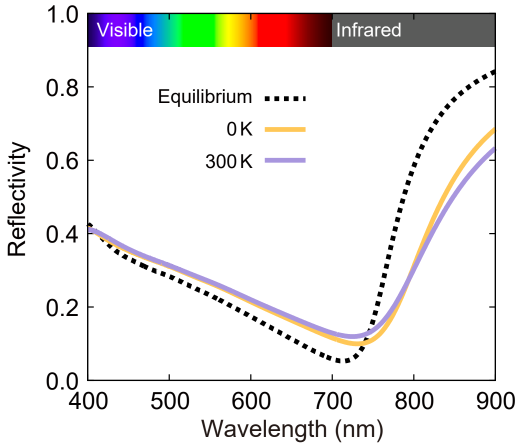

FIG. S5: **Quantum and thermal fluctuations effects on the electron-phonon coupling renormalised reflectivity of LuH_2.**

Our calculations including electron-phonon coupling are capable of describing the effects of both quantum fluctuations at 0 K and thermal fluctuations at finite temperature. To evaluate the relative contribution of quantum and thermal fluctuations, in Fig. S5 we compare

the reflectivity of LuH_2 obtained by fixing the nuclei at their equilibrium positions (no quantum nor thermal fluctuations), at 0 K (including only quantum fluctuations) and at 300 K (including quantum and thermal fluctuations). The difference between the equilibrium and 0 K curves quantifies the role of quantum fluctuations, which lead to a shift of the reflectivity minimum and to a decrease of the reflectivity in the infrared part of the spectrum, but the overall reflectivity shape does not change significantly. The difference between the 0 K and 300 K curves quantifies the role of thermal fluctuations, which make a significantly smaller contribution.

Supplementary Note 4. LUTETIUM-HYDROGEN BINARY SYSTEM

4.1. Crystal structures of LuH_2 and LuH_3

We have performed extensive structure searches for stoichiometries ranging from LuH_0 to LuH_3 and at multiple pressures. The results are summarised in the convex hull diagrams depicted in Fig. 2**a** in the main text. Here, we focus on the stoichiometric LuH_2 and LuH_3 as they are mostly relevant phases studied in the literature. We determine all competing structures in the pressure range 0-1000 kbar.

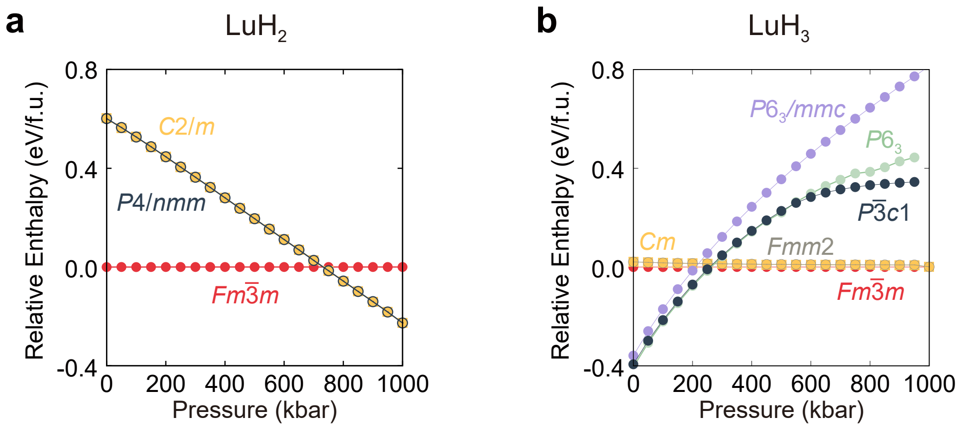

FIG. S6: **Relative enthalpy of LuH_2 and LuH_3.**

Figure S6 shows the relative enthalpy of the competing structures. For LuH_2, the ground state structure at 0 kbar has cubic $Fm\bar{3}m$ symmetry. A structure of $P4/nmm$ symmetry becomes more stable above 732 kbar. We note that there is a structure of space group $C2/m$ whose energy is almost degenerate with that of the $P4/nmm$ structure across the entire pressure range. In the main text, we report the pressure-driven reflectivity and colour changes of the $Fm\bar{3}m$ structure only. We report the reflectivity and colour of the $P4/nmm$ structure in Fig. S7.

For LuH_3, there are multiple competing phases in the pressure range 0-1000 kbar. The most important ones are the trigonal $P\bar{3}c1$ and the cubic $Fm\bar{3}m$ structures, as they are the most consistent with experimental observations. In the main text, we present the reflectivity and colour of $P\bar{3}c1$ at 0 and 100 kbar and $Fm\bar{3}m$ at 400 kbar according to the relative enthalpy. The full reflectivity and colour data of both structures over the entire pressure range of interest are presented in Fig. S8. However, we note that both of these structures are dynamically unstable at 0 kbar (see Fig. S15 for an example), and the most stable structure

at 0 kbar is the $P6_3$ structure, which is almost degenerate in energy with the $P\bar{3}c1$ structure. We note that the $P6_3$ structure is insulating up to a pressure of about 100 kbar. We also note that two structures with space groups Cm and $Fmm2$ are almost degenerate in energy with the cubic $Fm\bar{3}m$ structure over the entire pressure range of interest (see Fig. S9 for the reflectivity and colour of the $Fmm2$ structure).

4.2. Reflectivity and colour of LuH_2 and LuH_3

Multiple structure searching works have predicted a range of stable and metastable structures in the binary Lu-H system that may play a role in the reported superconductivity, and we report our own structure searching results in the main text. Here, we present the reflectivities and colours of the most important phases of LuH_2 (Fig. S7) and LuH_3 (Fig. S8).

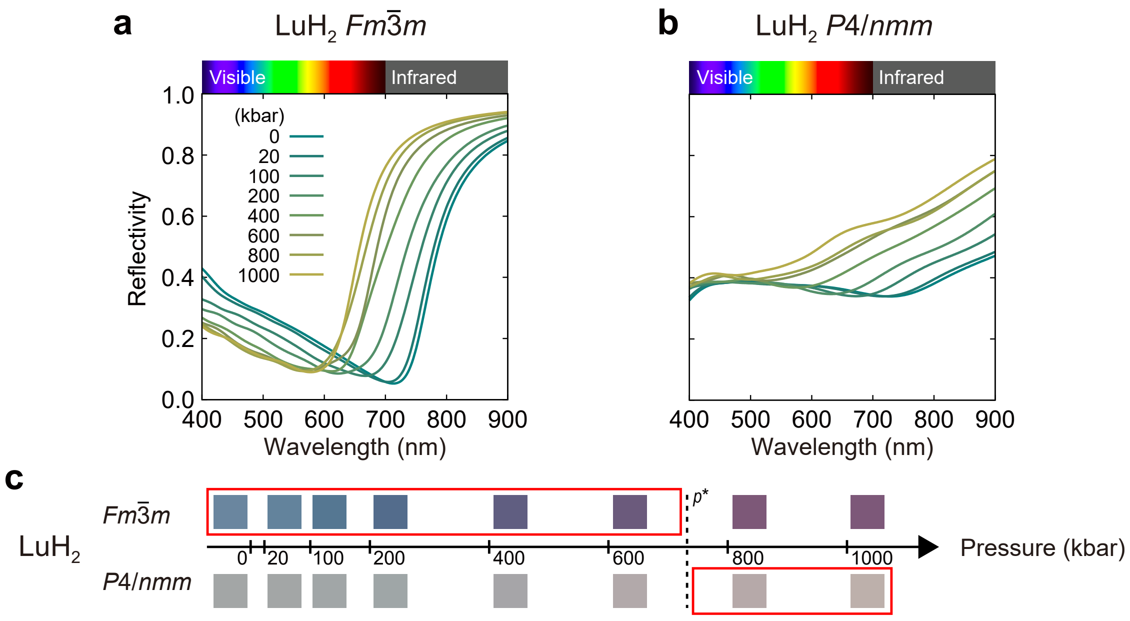

FIG. S7: **Reflectivity and colour of LuH_2.**

Additionally, we show the calculated reflectivities and colours for the LuH_3 structures with space groups $Fmm2$ and $P6_3/mmc$ in Fig. S9. Both structures exhibit a rather featurless reflectivity which leads to a light grey colour for $Fmm2$ LuH_3 and to a dark grey colour for $P6_3/mmc$ LuH_3. We have also calculated the reflectivity of the $C2/m$ LuH_2 structure (not shown), and it is very similar to that of the $P4/nmm$ structure, to which it is almost degenerate in enthalpy (Fig. S6).

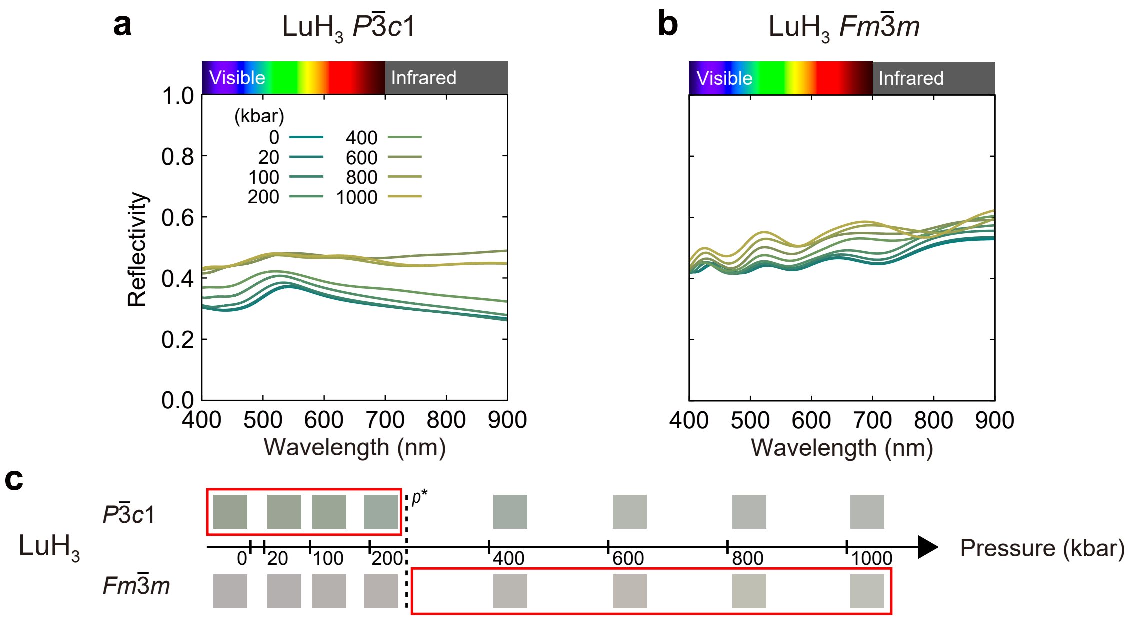

FIG. S8: **Reflectivity and colour of LuH_3.**

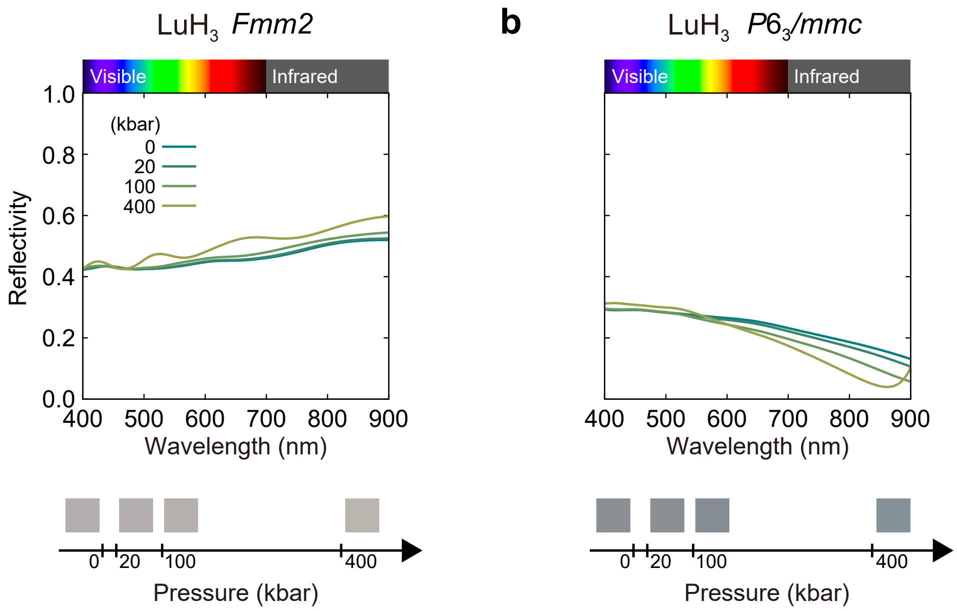

FIG. S9: **Reflectivity and colours of $Fmm2$ and $P6_3/mmc$ LuH_3 structures.**

4.3. Reflectivity and colour of nitrogen doped cubic LuH_2 and LuH_3

We establish hydrogen-deficient LuH_2 as the dominant phase responsible for the experimentally observed colour changes. However, multiple experimental reports include a small amount of nitrogen doping, and in this subsection we provide an overview of the interplay between nitrogen doping and colour in $LuH_{2-\delta}$ and $LuH_{3-\delta}$.

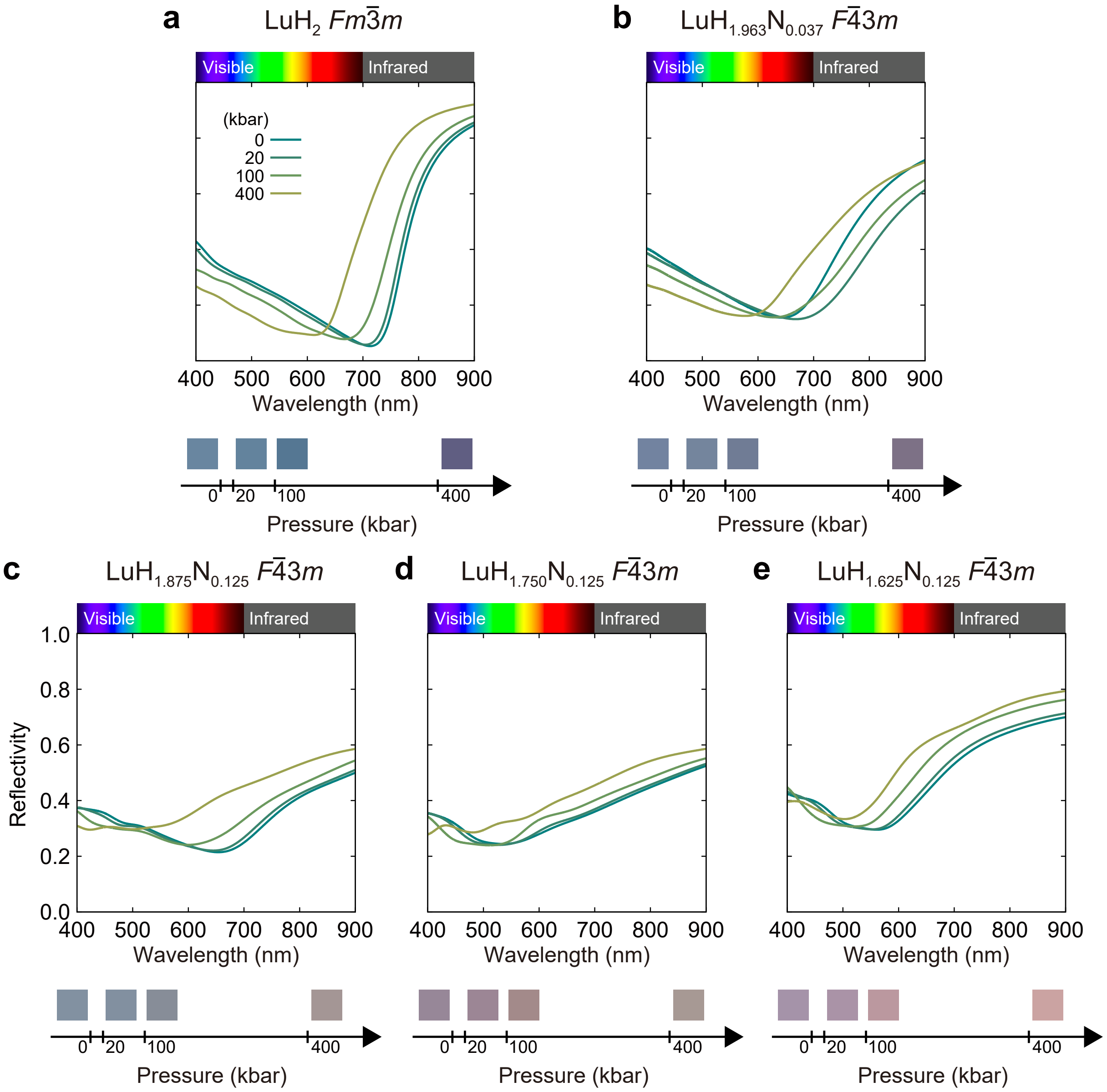

FIG. S10: **Reflectivity and colour of $LuH_{2-\delta}N_\epsilon$ structures.** $LuH_{1.963}N_{0.037}$ is simulated as a single hydrogen vacancy and a single nitrogen addition in a $3 \times 3 \times 3$ supercell. Other structures are simulated in a $2 \times 2 \times 2$ supercell.

We show the pressure-driven changes in the reflectivity and colour of nitrogen-doped

$LuH_{2-\delta}$ in Fig. S10. The effect of nitrogen doping is to flatten the reflectivity curves obtained with $LuH_{2-\delta}$, such that the resulting colours become more grey. As a result, we conclude that nitrogen doping also affects the reflectivity and colour of LuH_2, but the changes are secondary compared to those driven by hydrogen vacancies.

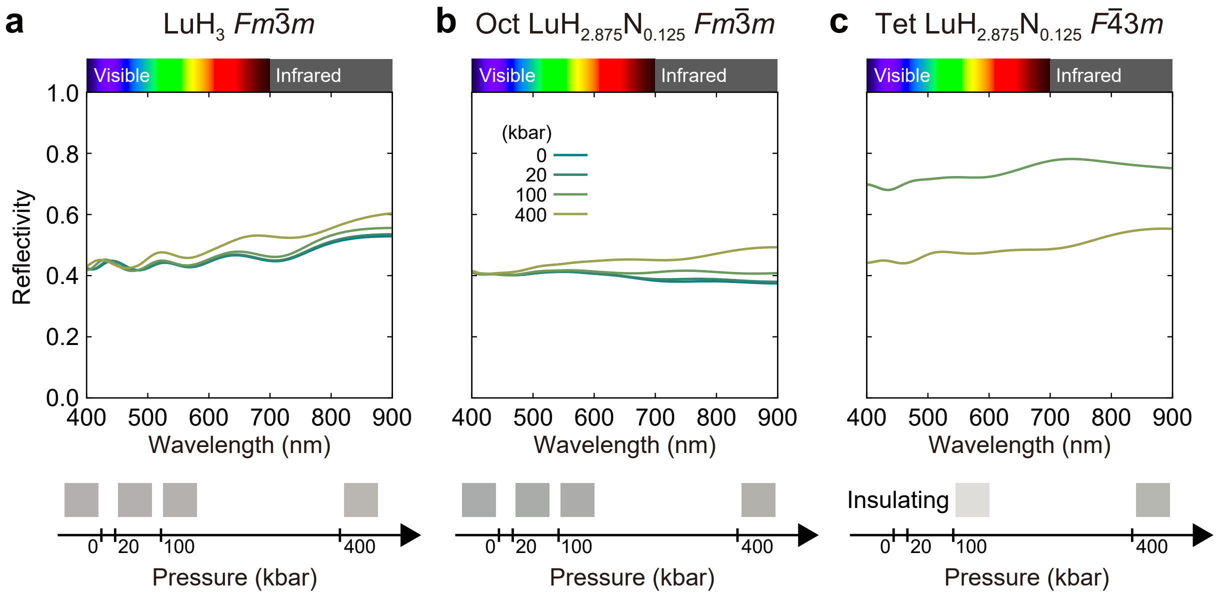

FIG. S11: **Reflectivity and colour of $LuH_{3-\delta}N_{\epsilon}$ structures.** The single hydrogen vacancy in LuH_3 is simulated in a $2 \times 2 \times 2$ supercell. In **b** and **c**, Oct and Tet denote the octahedral and tetrahedral sites for the hydrogen vacancy, respectively. In **c**, the structure is insulating at low pressures.

For completeness, we also show the pressure-driven changes in the reflectivity and colour of nitrogen-doped $LuH_{3-\delta}$ in Fig. S11. Nitrogen doping has a small effect in the reflectivities, which remain relatively flat leading to overall grey colours.

Supplementary Note 5. LUTETIUM-HYDROGEN-NITROGEN TERNARY SYSTEM

5.1. Crystal structures

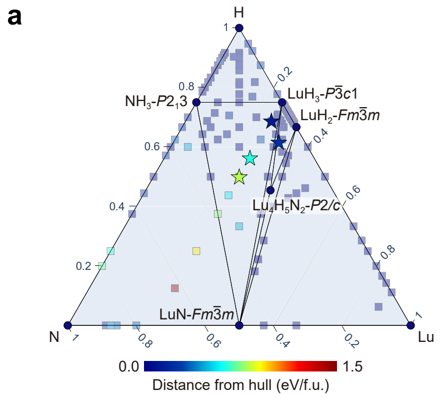

b

Compound	Space group	Distance from hull (meV/atom)			
		0 kbar	20 kbar	100 kbar	400 kbar
$Lu_4H_5N_2$	*P2/c*	0	0	0	0
Lu_4H_8N	$Pm\bar{3}m$	245	248	258	337
$Lu_4H_{11}N$	$Pm\bar{3}m$	289	275	237	181
$Lu_4H_9N_3$	$Pm\bar{3}m$	636	614	565	473
LuH_2N	$Fm\bar{3}m$	812	786	725	596

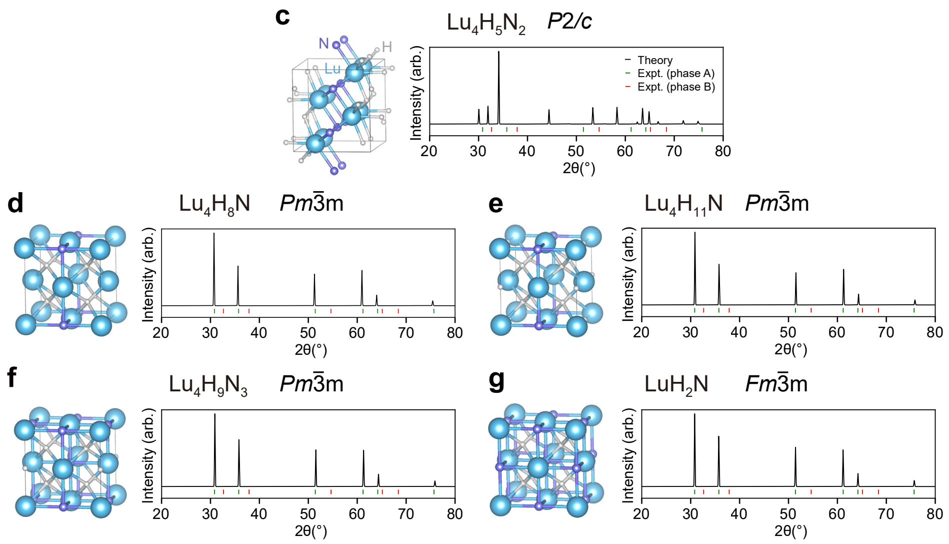

FIG. S12: **Convex hull diagram, energetics, and atomic structures of the lutetium-hydrogen-nitrogen ternary system. a.** Convex hull diagram for the Lu-H-N ternary system at ambient pressure. Dark blue circles indicate thermodynamically stable structures and metastable structures are shown as squares and stars. Stars indicate the cubic structures whose simulated X-ray diffraction (XRD) pattern is consistent with the experimental report [4], as shown in **d-g**. The colour scale represents the energy distance from the convex hull. **b.** Based on the calculated distance from the hull, we present the energetics of the five best Lu-H-N compounds including thermodynamically stable $Lu_4H_5N_2$ as a function of pressure. **c-g.** Atomic structures and XRD simluation results for **c** $Lu_4H_5N_2$, **d** Lu_4H_8N, **e** $Lu_4H_{11}N$, **f** $Lu_4H_9N_3$, and **g** LuH_2N. Except for the $Lu_4H_5N_2$ compound with $P2/c$ symmetry, Lu atoms form a fcc lattice. For the XRD similulations, experimental XRD data are displayed as green and red ticks, which are assigned as two different phases in Ref. [4].

We perform extensive crystal structure search in the full Lu-H-N ternary space (Fig. S12). We highlight that our structure searches [5] are the only ones of all those published that identify a stable ternary compound $P2/c$ $Lu_4H_5N_2$ at ambient pressure. We have selected multiple ternary Lu-H-N compounds $Pm\bar{3}m$ Lu_4H_8N, $Pm\bar{3}m$ $Lu_4H_{11}N$, $Pm\bar{3}m$ $Lu_4H_9N_3$, and $Fm\bar{3}m$ LuH_2N that are not on the convex hull but whose simulated X-ray diffraction data is consistent with experimental reports. The calculated colours for these compounds are shown in Fig. S13.

5.2. Reflectivity and colour

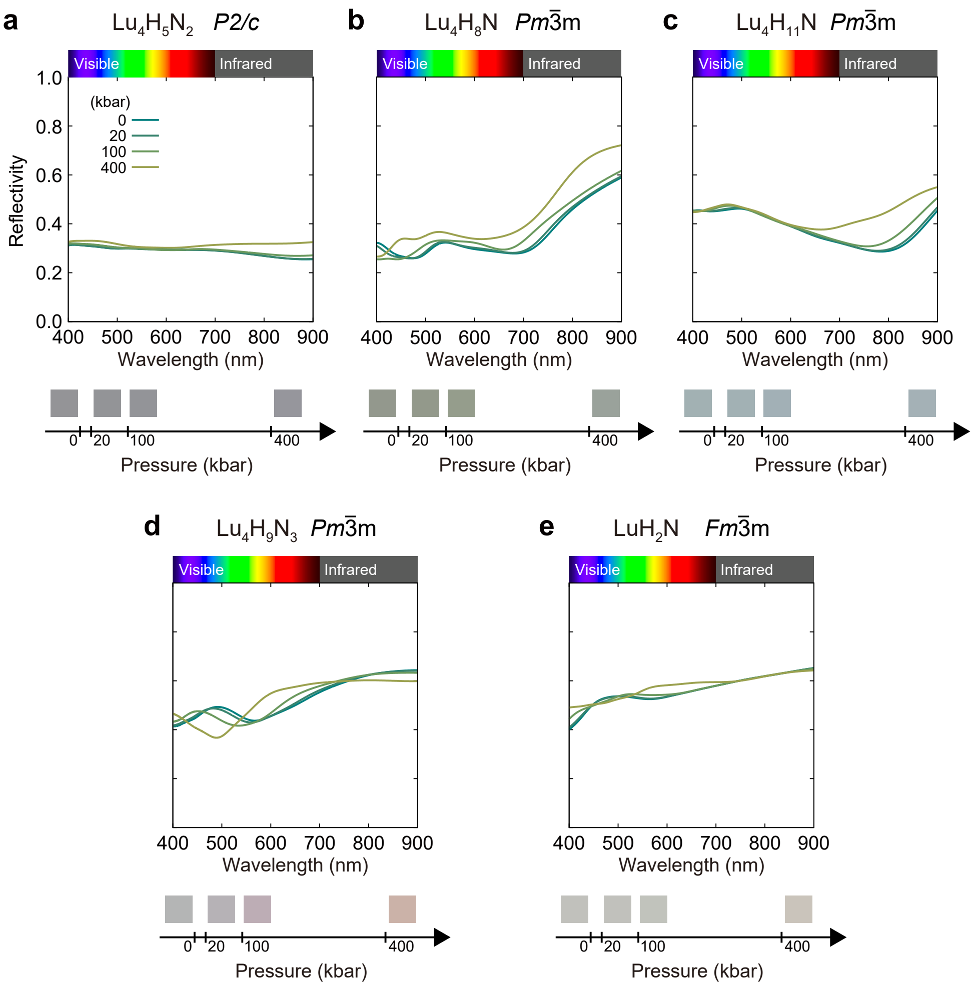

FIG. S13: **Pressure dependence of reflectivity and colour of the lutetium-hydrogen-nitrogen ternary system.**

Multiple structure searching works have predicted a stable and multiple metastable struc-

tures in the ternary Lu-H-N system that may play a role in the reported superconductivity. $Lu_4H_5N_2$ is the only structure predicted to be thermodynamically stable in the Lu-H-N ternary system, and we show its reflectivity and colour as a function of pressure in Fig. S13**a**. Its reflectivity in the visible range is small and constant, leading to an overall dark grey colour. We also display the reflectivities and colours for four additional metastable structures of stoichiometries Lu_4H_8N, $Lu_4H_{11}N$, $Lu_4H_9N_3$, and LuH_2N in Figs. S13**b-e**. These structures are metastable but all have cubic symmetry, which has been identified experimentally as the relevant symmetry. Their reflectivities are relatively constant across the visible range of the spectrum, leading to overall grey colours with a slight blue tone in $Lu_4H_{11}N$ at ambient pressure.

Supplementary Note 6. PHONON DISPERSION

Regarding the dynamical stability of the compounds, we have confirmed that the pure LuH_2 and hydrogen deficient $LuH_{2-\delta}$ structures, depicted in photorealistic rendering in Fig. 3 in the main text, exhibit dynamic stability at the harmonic level, as displayed in the phonon dispersion in Fig. S14. The phonon band structures show a large gap separating the low-frequency acoustic modes from the high frequency optical modes. This large gap is expected in binary compounds with a large mass difference between the elemental components, with hydrogen having a relative atomic mass of 1.008 and lutetium of 174.967.

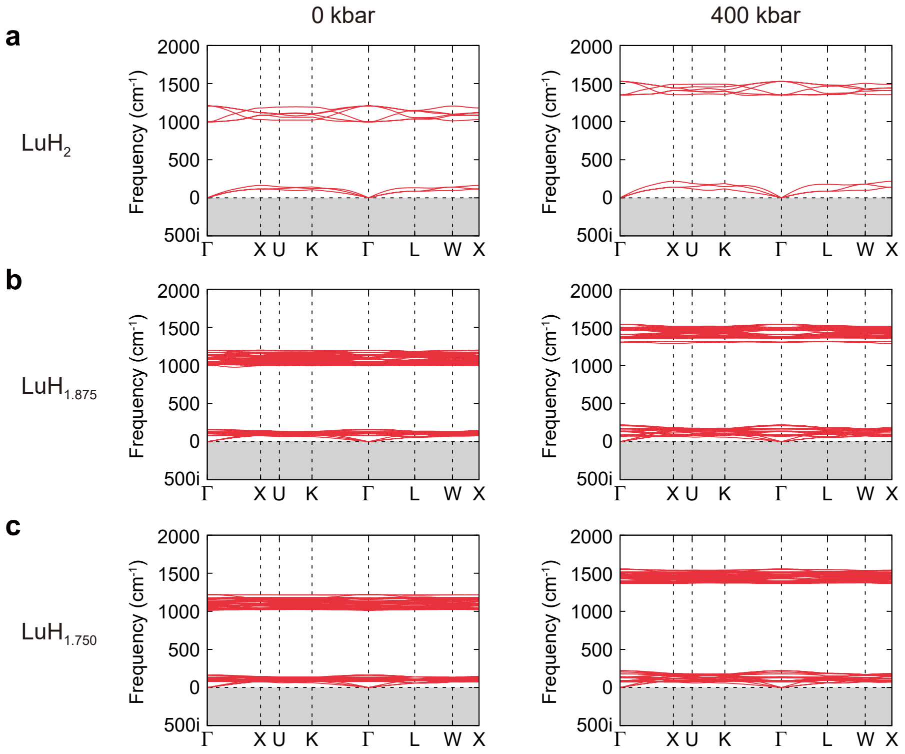

FIG. S14: **Phonon dispersion of pure and hydrogen-deficient lutetium dihydrides at** 0 **and** 400 **kbar.**

We have also confirmed the dynamical stability of multiple other structures (e.g. LuH in Fig. S15**a**). However, we note that cubic LuH_3, proposed as the phase responsible for superconductivity in the original Nature paper by Dias and co-workers, is dynamically unstable at the harmonic level (Fig. S15**b**). This has also been reported by other works.

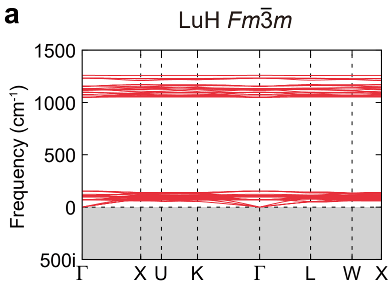

FIG. S15: **Phonon dispersion of lutetitum monohydride and trihydride.** Results are shown for **a** LuH in the $Fm\bar{3}m$ structure and **b** LuH_3 in the $Fm\bar{3}m$ structures.

Supplementary Note 7. PHONON-MEDIATED SUPERCONDUCTIVITY

There have been several reports of phonon-mediated superconductivity calculations in a range of stable and metastable compounds of the Lu-H-N system. Interestingly, none of these calculations find superconducting critical temperatures near room temperature. Experimentally, the superconducting phase is claimed to only exist when the sample exhibits a pink colour. Given our discovery that the pink phase of lutetium hydride only exists in hydrogen-deficient LuH_2, we estimate the phonon-mediated superconducting critical temperature of $LuH_{1.875}$ and $LuH_{1.750}$ as a function of pressure. Figure S16 shows the isotropic Eliashberg function $\alpha^2F(\omega)$ including the pink colour phases of $LuH_{1.875}$ at 400 kbar and $LuH_{1.750}$ at 20 kbar. The corresponding superconducting critical temperatures for $LuH_{1.875}$ ($LuH_{1.750}$) at 20, 100, and 400 kbar are 0.19 K (0.04 K), 0.16 K (0.03 K), and 0.33 K (0.06 K), respectively.

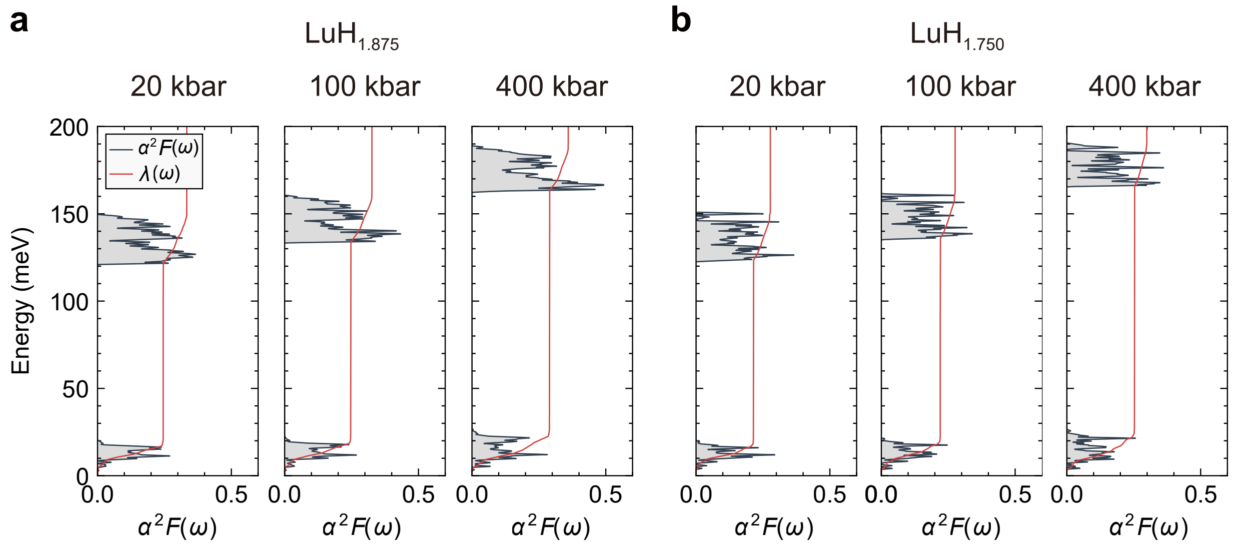

FIG. S16: **Isotropic Eliashberg function of the hydrogen-deficient $LuH_{1.875}$ and $LuH_{1.750}$ at multiple pressures.**

Supplementary Note 8. DETAILS OF PHOTORELIASTIC RENDERING

8.1. Benchmark tests on Lu and LuH_2

We have described the computational methodology used to calculate colour in Methods, which was originally developed and tested by Prandini and co-workers [6]. They applied the methodology to a wide variety of metallic systems, including 18 elemental metals (e.g. Au, Cu, Al), intermetallic compounds (e.g. $AuAl_2$, PdIn), and solid solutions (e.g. Au-Ag solid solutions), in all cases demonstrating remarkable agreement between theory and experiment. Therefore, the computational method we use has been thoroughly validated for many compounds. As an additional validation, in our work we have tested the calculation of the colour of ambient conditions LuH_2 and Lu, and again our results are in good agreement with experiment (Fig. S17).

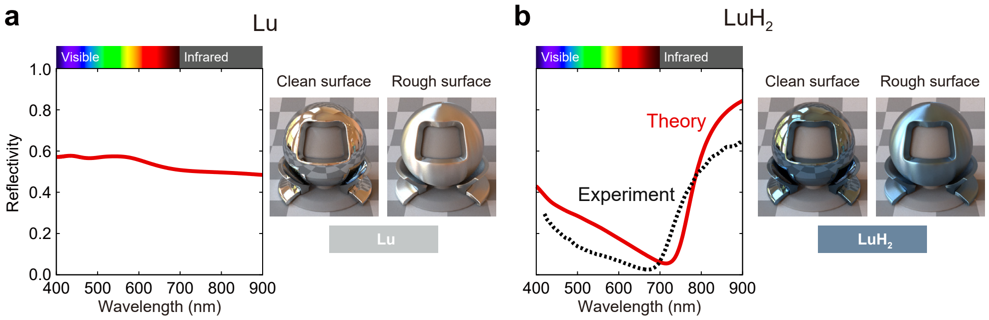

FIG. S17: **Reflectivity and colour of Lu and LuH_2.** Here, we present two photorealistic rendering images for each system with different surface roughness conditions (clean or rough surface) as implemented in the MITSUBA 3 renderer [7]. The photorealistic rendering of the Lu metal shows a silvery white colour, in good agreement with experiment [8].

8.2. Surface roughness effect

Figure S18 illustrates the blue colour of LuH_2 using different models for the surface roughness, which shows the type of change that one may expect between different samples. We find that the surface effect does not change the colour. We highlight that the key quantity to describe the optical response of materials is the dielectric function, and we include all calculated dielectric functions as Supplementary Data to enable readers to evaluate the optical response for distinct setups.

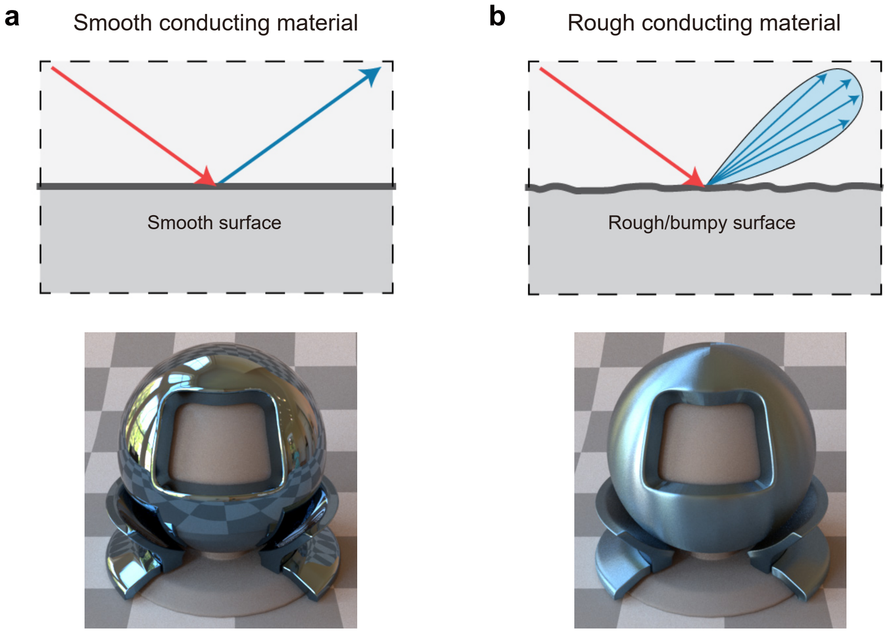

FIG. S18: **Surface roughness effect.** Photorealistic rendering images for LuH_2 with different surface roughness conditions (clean or rough surface) as implemented in the Mitsuba 3 renderer [7]. The images in upper panels are taken from this webpage on the Mitsuba 3 renderer website. For **b**, we use roughness values of $\alpha_u = 0.05$ and $\alpha_v = 0.3$.

8.3. Choice of exchange-correlation functional

We note that the brightness of the colour can change if we use different exchange-correlation functionals for the DFT calculations. For example, Figure S19 shows DFT+U results compared with DFT, with the former exhibiting brighter orange colours and a transition to a red phase in pure LuH_2. Despite these small change depending on the calculation details, the overall conclusions of our work remain unchanged.

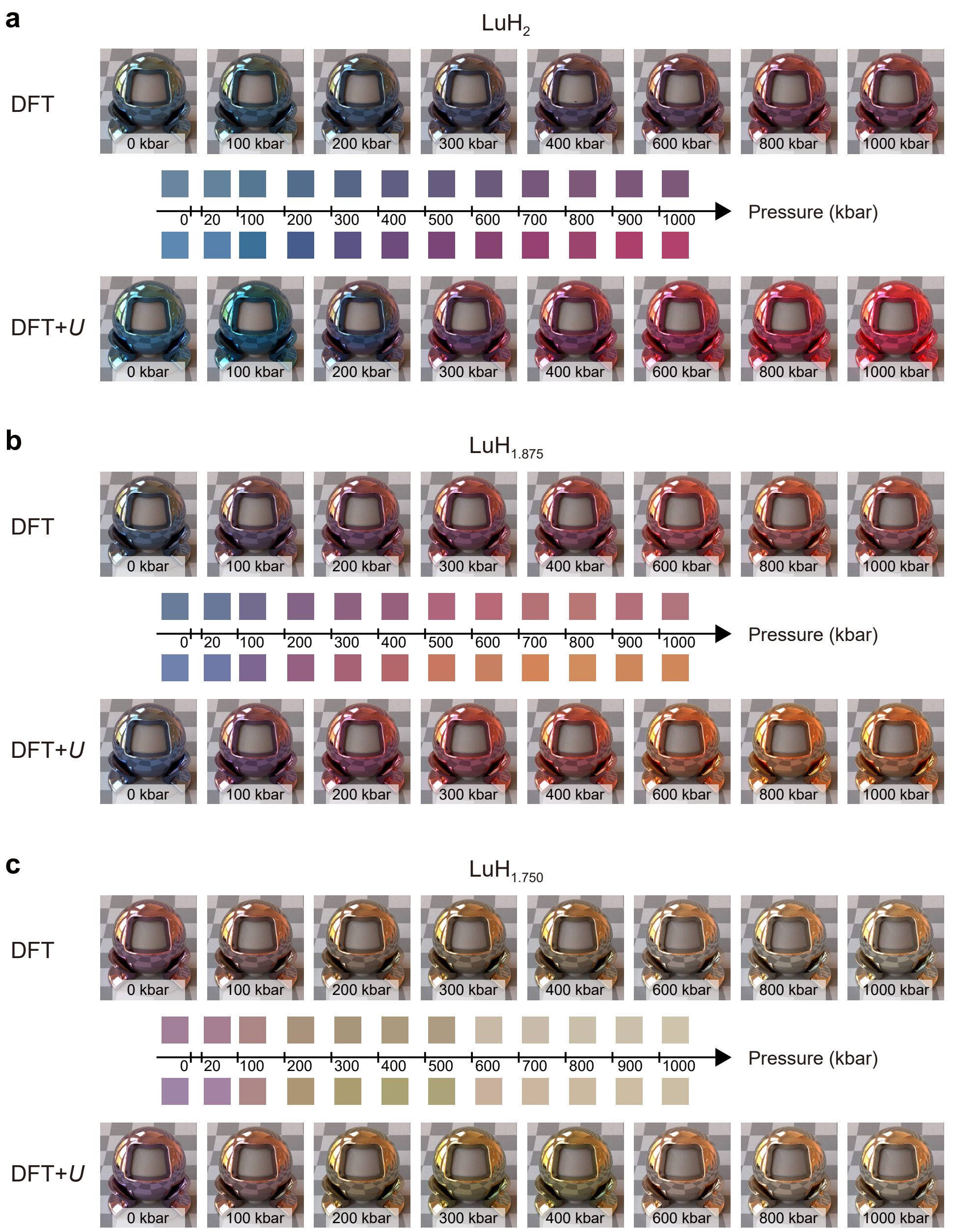

FIG. S19: **Comparison between DFT and DFT+U colour results. a-c.** Colour and photorealistic rendering of **a** LuH_2, **b** $LuH_{1.875}$ and **c** $LuH_{1.750}$ as a function of pressure. We use $U = 3\,\text{eV}$ on Lu d orbitals.

Supplementary Note 9. CONVERGENCE TESTS ON REFLECTIVITY

We have tested various convergence parameters on the calculation of reflectivity, and found that an energy cutoff of 400 eV and a **k**-point grid size of 40 × 40 × 40 are fully converged. Convergence test details are presented in Fig. S20.

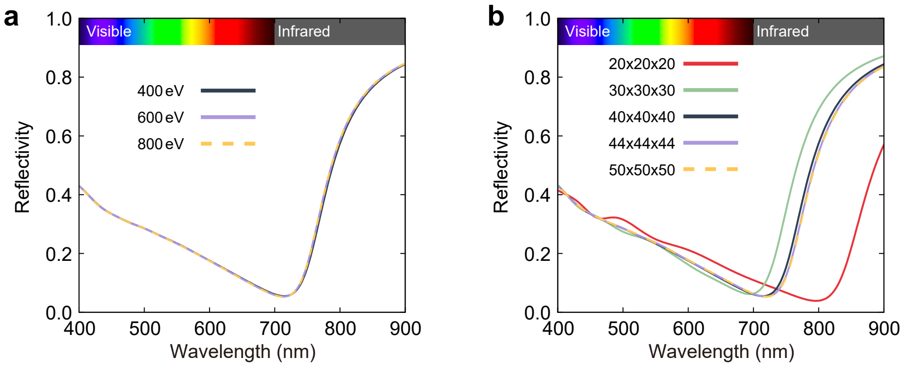

FIG. S20: **Convergence tests on reflectivity.**

[1] Williams, F. Theoretical low temperature spectra of the thallium activated potassium chloride phosphor. *Phys. Rev.* **82**, 281–282 (1951). URL https://journals.aps.org/pr/pdf/10.1103/PhysRev.82.281.2.

[2] Lax, M. The Franck-Condon principle and its application to crystals. *J. Chem. Phys.* **20**, 1752–1760 (1952). URL https://aip.scitation.org/doi/pdf/10.1063/1.1700283?class=pdf.

[3] Monserrat, B. Vibrational averages along thermal lines. *Phys. Rev. B* **93**, 014302 (2016). URL https://link.aps.org/doi/10.1103/PhysRevB.93.014302.

[4] Dasenbrock-Gammon, N. *et al.* Evidence of near-ambient superconductivity in a N-doped lutetium hydride. *Nature* **615**, 244 (2023). URL https://doi.org/10.1038/s41586-023-05742-0.

[5] Ferreira, P. P. *et al.* Search for ambient superconductivity in the Lu-N-H system. *Nature Communications* **14**, 5367 (2023). URL https://doi.org/10.1038/s41467-023-41005-2.

[6] Prandini, G., Rignanese, G.-M. & Marzari, N. Photorealistic modelling of metals from first principles. *npj Computational Materials* **5**, 129 (2019). URL https://doi.org/10.1038/s41524-019-0266-0.

[7] Jakob, W. *et al.* Mitsuba 3 renderer (2022). Https://mitsuba-renderer.org.

[8] Zhang, S. *et al.* Electronic and magnetic properties of Lu and LuH_2. *AIP Advances* **13**, 065117 (2023). URL https://doi.org/10.1063/5.0153011.
